# High‐Throughput Culture and DNA Isolation Methods for *Aspergillus fumigatus*

**DOI:** 10.1002/cpz1.70112

**Published:** 2025-04-02

**Authors:** Francisca C. Reyes Márquez, Ben Auxier, Bo Briggeman, Eveline Snelders

**Affiliations:** ^1^ Laboratory of Genetics, Plant Sciences Group Wageningen University Wageningen The Netherlands; ^2^ Infectious Disease Research, Diagnostics and Laboratory Surveillance, Centre for Infectious Disease Control National Institute for Public Health and the Environment Bilthoven The Netherlands

**Keywords:** 96‐well format, *Aspergillus fumigatus*, genotyping, large‐scale fungal screening, phenotyping

## Abstract

*Aspergillus fumigatus* is an opportunistic human fungal pathogen that is widely spread in the environment. The need to screen a large number of environmental or clinical isolates for phenotypic and/or genotypic purposes can be challenging and time consuming when using the protocols that are currently available. We have developed a novel approach that allows one to inoculate cultures, grow individual *A. fumigatus* isolates, and subsequently isolate DNA of sufficient quality for PCR and/or whole genome sequencing, all in a 96‐well format. Compared to currently available methods, our protocols reduce both cost and labor significantly and are compatible with any experimental setup that uses a 96‐well format. © 2025 The Author(s). Current Protocols published by Wiley Periodicals LLC.

**Basic Protocol 1**: 96‐well‐format mini slant culture and preparation of spore suspension for *Aspergillus fumigatus*

**Basic Protocol 2**: PCR‐quality DNA isolation

**Basic Protocol 3**: High‐purity DNA isolation after mini mycelial mat culture

## INTRODUCTION

*Aspergillus fumigatus* is an opportunistic human pathogen found widely in the environment. It is a saprobic fungus that thrives on dead plant material and produces large amounts of airborne spores, which are spread by wind‐borne dispersal and have a broad geographic distribution. *A. fumigatus* can cause a range of diseases, with invasive aspergillosis being the most lethal manifestation, affecting immunocompromised patients (Latgé & Chamilos, 2019). Aspergillus diseases rely heavily on the use of the triazole class of antifungal compounds. This class is used not only in medicine but also for crop protection against plant pathogenic fungi. Environmental exposure of *A. fumigatus* to triazole residues in plant waste heaps has been identified as a major risk factor for antifungal resistance selection (Snelders et al., 2012; Verweij et al., 2009). Research now aims to understand the exact circumstances of antifungal resistance selection as well as the routes of transmission from the environment to humans. Therefore, multiple efforts are being made to screen broad geospatial regions (Shelton et al., 2023) and record the presence of *A. fumigatus* to identify antifungal‐resistant genotypes by molecular techniques (Kortenbosch et al., 2022; Rhodes et al., 2022). Currently, available methods for growing *A. fumigatus* and isolating DNA are not high throughput and are often time consuming. Furthermore, as a model species, marker‐free gene editing mediated by endonucleases like Cas9 is often used with *A. fumigatus*, which, due to low efficiency, sometimes requires the selection of large numbers of colonies to identify the desired edited genotype (van Rhijn et al., 2020). Such projects can require molecular screening of hundreds of samples, necessitating significant amounts of time and labor for the sample processing and downstream molecular analysis. This also requires considerable space usage for incubators and storage and high use of consumables that often, unfortunately, limit the scale of feasible experiments. To address the need to screen genotypically at large scale, tools need to be developed to enable high‐throughput screening.

**Figure 1:**
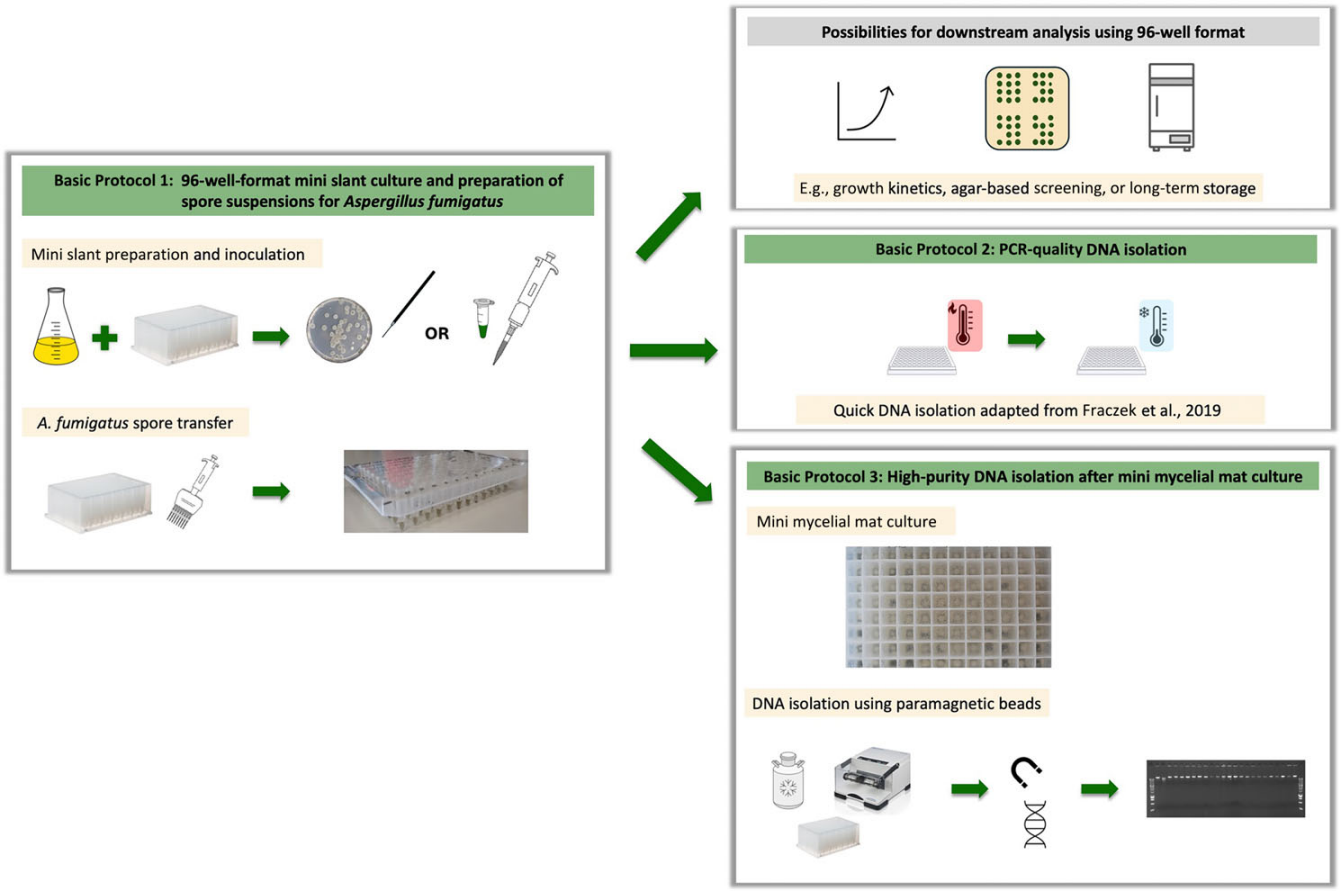
Protocol overview. The methods described in this study aim to culture *A. fumigatus* in a 96‐well format and conduct different downstream protocols depending on the needs of the desired downstream analysis. Basic Protocol 1, involving 96‐well‐format mini slant culture for *A. fumigatus* (left panel), describes the mini slant preparation and inoculation from a single colony, grown on a plate, or pre‐existing spore suspensions, all in a 96‐well plate. From the master plate generated at this point, immediate applications, such as growth kinetics assays or agar‐based screening, or long‐term storage is possible (upper right panel). Basic Protocol 2, describing PCR‐quality DNA isolation (middle right panel), provides an updated version of the quick DNA heat‐shock isolation protocol from a prior Current Protocols article (Fraczek et al., 2019). Using this protocol, sufficient template for PCR genotyping can be obtained to perform amplicon size separation (gel electrophoresis), Sanger sequencing, and/or Nanopore amplicon sequencing. Basic Protocol 3, describing high‐purity DNA isolation, illustrates how to grow mini mycelial mats in a 96‐well format and how to isolate high‐molecular‐weight DNA using paramagnetic beads (lower right panel). The genomic DNA obtained using this protocol can be employed for library preparation for whole genome sequencing.

Here, we describe a novel method to facilitate high‐throughput *A. fumigatus* screening by using a 96‐well mini slant format (Basic Protocol 1) that produces sufficient input material from asexual spores to be used for at least two different genomic DNA isolation methods (Basic Protocols 2 and 3) or for use in other experimental work in a 96‐well format. In our approach, each of the 96‐well mini slant deep wells can directly be inoculated with an *A. fumigatus* isolate from single spore–grown colonies (e.g., from Petri dish culture plates) or from a pre‐made spore suspension. After harvesting spores from the 96‐well mini slant deep‐well plate in a suspension, also in a 96‐well format, these newly prepared spore suspensions can be used for both an already‐existing heat‐shock protocol to obtain PCR‐quality DNA (see Current Protocols article: Fraczek et al., 2019), for which some modifications were made (Basic Protocol 2), and for an adapted protocol that uses paramagnetic beads to obtain high‐quality DNA, which can be used for next‐generation sequencing (NGS; Basic Protocol 3) (Fig. 1). The 96‐well mini slant deep‐well plate protocol can also be useful for other high‐throughput phenotypic screening methods following a 96‐well format.

## STRATEGIC PLANNING

Our 96‐well format for *A. fumigatus* culture offers flexibility for different downstream analyses (Fig. 1). For DNA isolates, determine the needs and scope of your project to decide which protocol you desire to use after Basic Protocol 1. This could be either Basic Protocol 2 for PCR‐quality DNA isolation or Basic Protocol 3 for high‐purity DNA isolation.

*CAUTION: A. fumigatus* is a biosafety level class 2 organism. Follow all appropriate national guidelines and regulations for the use and handling of pathogenic microorganisms. Preparation of plates, inoculation, and harvesting of *A. fumigatus* in the 96‐well plates should be carried out in a biosafety cabinet to reduce the chance of (cross‐)contamination, and the appropriate safety regulations to work with this fungus should be followed.

## Basic Protocol 1 96‐WELL‐FORMAT MINI SLANT CULTURE AND PREPARATION OF SPORE SUSPENSION FOR *Aspergillus fumigatus*

In this protocol, mini slants are produced in a 96‐deep‐well plate format, aiming to culture many *A. fumigatus* isolates while yielding enough spores to perform amplicon screening (Basic Protocol 2), isolate high‐quality genomic DNA (Basic Protocol 3), or perform other 96‐well screening methods and finally to make glycerol stocks for storage in the – 80°C freezer. Here, we describe how to make the mini slants, how to carry out their inoculation, and how to harvest the spores after incubation.

### Materials

Malt extract agar (MEA) medium (see recipe), 55° to 60°C
*A. fumigatus* spore suspension or unique colony‐forming unit from agar plate
PBS‐0.05% Tween‐20 buffer (see Current Protocols article: Fraczek et al., 2019; see recipe)

96‐deep‐well plate, 2 ml, pyramid bottom, 44‐mm height, sterile (Greiner, cat. no. 780271)
Multichannel reagent reservoirs, sterile
Multichannel pipet (100 to 1000 µl)
2‐mm cotton swabs (Heinz Herenz Hamburg, cat. no. 31032238) or tungsten inoculation needle
96‐deep‐well‐format lid, sterile
Micropore tape
37°C incubator
Plate shaker (Heidolph, Titramax 1000) at 37°C
96‐well PCR plate, sterile
96‐well PCR seal

1 Pour warm (55° to 60°C) MEA medium into a sterile multichannel reagent reservoir.
2 Hold the sterile 96‐deep‐well plate horizontally while adding 600 µl warm MEA medium, using a multichannel pipet to produce a slanted surface using a specific “dragging” manner (see Video 1). Let it solidify in horizontal position for ∼20 min on a uniformly sloped surface.
  If mini slants do not solidify on a slope, refer to Table 1 for possible causes and solutions.

**Video 1:** Overview of the pipeteting procedure to prepare tilted MEA mini‐slants in a 96‐well plate. The key to maximizing the surface for inoculation is the dragging motion during pipetting the agar [0:12] and letting the agar solidify while the container is tilted [0:35].

**Table 1:** Troubleshooting Guide for Mini Slant *A. fumigatus* Culture and Downstream Applications

Problem	Possible cause	Solution
Mini slants do not show a slope	Cold medium was used	Keep the medium at 55‐60°C until the moment you are ready to put it on the plate.
	Agar concentration is too high (>1.5%)	Reduce the agar concentration to 1.2%. Agar is a natural product, and properties may vary across brands and/or production batches.
There is no growth on the slant in inoculated wells	Needle was too hot when harvesting the colony	Make sure to cool down the needle before touching the colony, either by waiting or by briefly touching the PBS solution or an empty MEA plate.
	There was no moisture on the needle when harvesting the colony	Dip the needle in PBS‐Tween or in MEA before touching the colony.
Spore suspension is visibly still clear after harvesting	The spores were not efficiently resuspended after addition of PBS‐Tween	Slightly increase the speed of the plate shaker.
		Use a multichannel pipet (100‐1000 µl) set to 200 µl to slowly pipet the spore suspension up and down. This should release the spores.
		Mycelial mat production can be inhibited by excess surfactant. If mycelial mats do not develop in 16‐24 hr, reducing surfactant in the solution may improve results.
Nonexistent or extremely low yield of genomic DNA	Mycelial mat was not properly ground	Increase the time of the grinding to 1 min when using the Retsch Mixer Mill.

**Figure 2:**
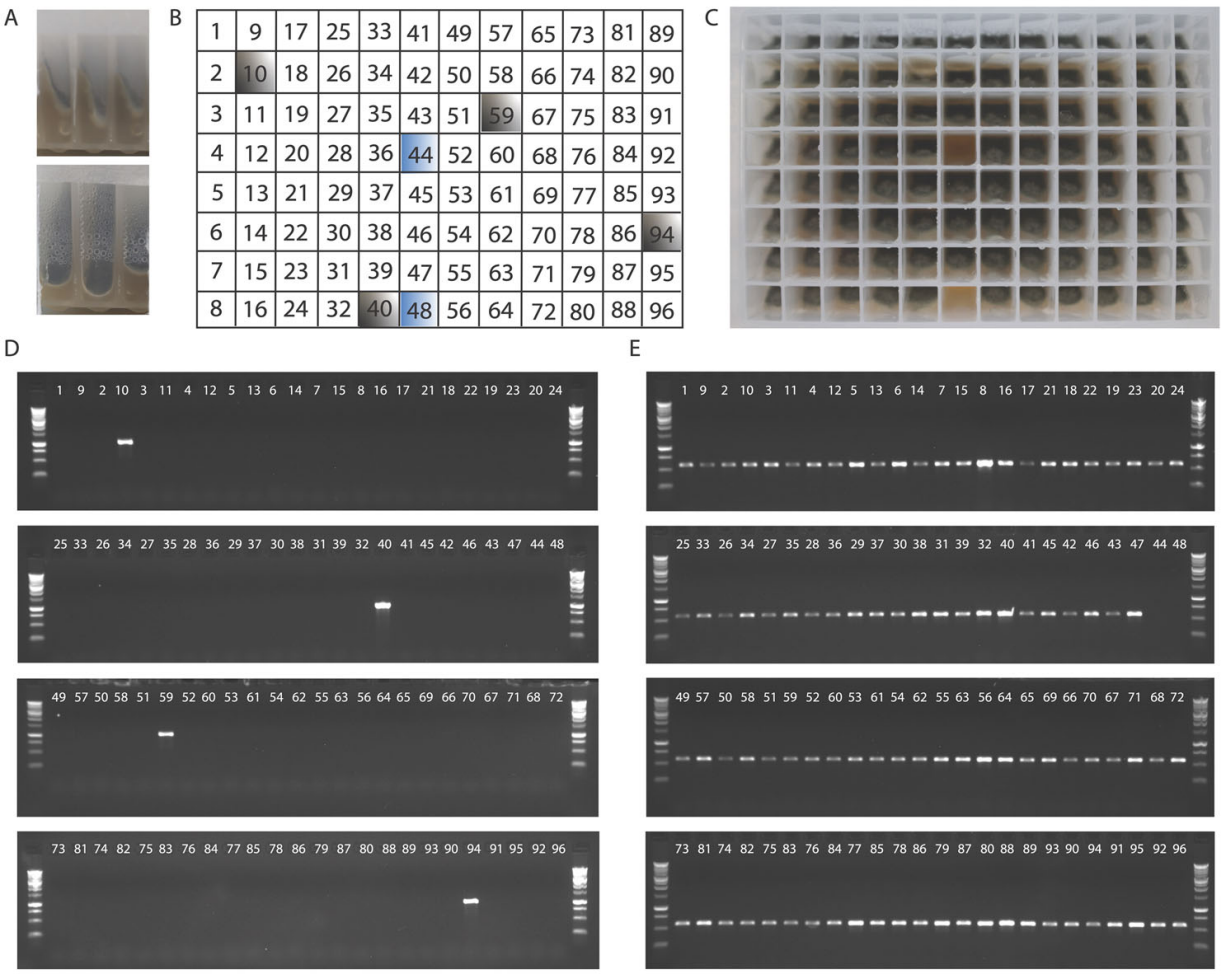
Mini slant growth and cross‐contamination control plate. By using a deep (2‐ml) 96‐well plate, we generate mini slants that can produce sufficient spores for multiple downstream procedures. *A. fumigatus* is inoculated on the lower portion of the agar using a tungsten needle if the input is a unique colony‐forming unit from a cultured agar plate or with a pipet in the case of spore suspensions. (**A**) After 48 hr of culture at 37°C, *A. fumigatus* has grown on the surface of the mini slant (the top panel shows the side view of the 96‐well plate, and the lower panel shows the front view), and spores are observed and ready to be harvested. To rule out the possibility of cross‐contamination between adjacent wells during the inoculation or culturing of *A. fumigatus*, we set up a control plate containing different inocula from which PCR‐quality genomic DNA can be extracted to determine possible contamination. (**B**) Plate arrangement for wells inoculated with wild‐type (AfIR974) *A. fumigatus* spores (white boxes), stable transformed *A. fumigatus* carrying the *hph* gene (gray boxes), and uninoculated negative‐control wells (blue boxes). (**C**) Fungal growth after 48 hr of incubation at 37°C. All the inoculated wells (94) show spores, except for negative‐control wells 44 and 48. Spores are collected, and a heat‐shock protocol is performed to obtain PCR‐quality DNA. (**D**) Gel images visualizing the PCR amplificons of the *hph* gene using primers targeting the coding region of *hph* (product size 1020 bp). Samples were loaded in alternating sample order due to spacing between the tips of the used multichannel pipet; this spacing may vary across brands. Only the four stable transformants displayed a *hph*‐positive genotype, indicating no detectable cross‐contamination between the inoculated wells. (**E**) PCR control to detect genomic DNA from *A. fumigatus*. A partial region from the gene encoding for calmodulin (*cmd*) was amplified using specific primers. All the 94 wells inoculated with *A. fumigatus* showed a band of the expected size (550 bp). The two growth‐control wells (44 and 48) showed no visible band, indicating absence of growth of *A. fumigatus* in these wells.

3 Inoculate each of the mini slants using 1 to 2 µl *A. fumigatus* spore suspension, or a tungsten inoculation needle if the intitial input is a unique colony forming unit (dry spores) from a cultured agar plate.
  Narrow cotton swabs (2 mm) can also be used but must be handled with care to not overload them with spores that can spread to neighboring mini slants.
  This step requires training, and therefore, it is important to practice inoculating mini slant plates and leaving negative‐control wells empty. Only when the negative controls remain negative after incubation is one ready to use this approach. Do not let yourself be discouraged if you fail on the first attempt; the learning curve is usually very steep.
  If no growth is present on the slants at all, refer to Table 1 for possible causes and solutions.
4 Once the plate has been inoculated, cover it with a sterile 96‐deep‐well‐format lid and secure it with a single layer of micropore tape.
5 Place in a 37°C incubator and incubate for 36 to 48 hr until green spores are visible (Fig. 2A).

6 To prepare the spore suspensions after culturing, first pour PBS‐0.05% Tween‐20 buffer into a sterile multichannel reagent reservoir.
7 Carefully and slowly add 600 µl PBS‐0.05% Tween‐20 buffer to each well of the plate from step 5 using the multichannel pipet, aiming to gently hit the wall of each well, and not the slant itself, to prevent the spores from rising and contaminating neighboring wells.
8 Put the sterile lid back on the plate, secure it with micropore tape, and incubate for 10 min at 500 rpm on a plate shaker at 37°C.
  After this time, the spores in each well should be fully in suspension and without cross‐contamination.
  This step can be done at room temperature or at 37°C, depending on the availability of a shaker at 37°C. If the solution is clear (meaning that not many spores are successfully in suspension) after the incubation, it is possible to resuspend the spores by carefully pipetting up and down before continuing to the next step. If the suspension does not show a dark‐green color but remains clear, refer to Table 1 for possible causes and solutions.
9 Transfer ≤150 µl from each well into a sterile 96‐well PCR plate using a multichannel pipet. After tightly closing the plate with a 96‐well PCR seal, store the master plate containing 96 individual genotypes at 4°C until further use (see Basic Protocols 2 and 3).
  We have used the spore suspensions obtained at this point to either perform modified quick genomic DNA isolation (see Current Protocols article: Fraczek et al., 2019) (see Basic Protocol 2) or isolate high‐purity genomic DNA by solid‐phase reversible immobilization (SPRI) using carboxyl‐coated paramagnetic beads (see Basic Protocol 3).

## Basic Protocol 2 PCR‐QUALITY DNA ISOLATION

Fraczek et al. (2019) describe a fast and reliable protocol to isolate genomic DNA from *A. fumigatus* spore suspensions. Here, we use that protocol as part of our 96‐well‐format method, with modification to allow it to be used without direct access to a –80°C freezer to further simplify and speed up the process. We use this modified heat‐shock protocol to generate a genomic DNA template that, after PCR amplification, can be used for agarose gel electrophoresis, Sanger sequencing, and/or Nanopore DNA amplicon sequencing.

### Materials

Spore suspension (in master plate; see Basic Protocol 1)

96‐well PCR plate, sterile
Multichannel pipet (100 to 1000 µl)
Thermal cycler or 95°C water bath
96‐well‐plate centrifuge

1 Take 30 to 100 µl spore suspension (in master plate; see Basic Protocol 1) and transfer to a sterile 96‐well PCR plate using a multichannel pipet.
2 Incubate for 15 min at 95°C using a thermal cycler or a water bath.
3 Transfer immediately to a –80°C freezer and incubate for ≥10 min.
  We have tested and validated that immediate transfer and incubation of the plate for 30 min in a –20°C freezer or 1 hr on ice is also enough to efficiently heat‐shock the spores.
4 If frozen, thaw to room temperature.
5 Centrifuge the plate for 30 s at 1000 × *g* in a 96‐well‐plate centrifuge to ensure that all spores are at the bottom of the wells.
6 Use 1 or 3 µl of the supernatant from the heat‐shocked spore suspension for PCR, depending on the final PCR volume (10 or 25 µl, respectively).
  Avoid transferring the spores and fungal debris when taking the supernatant for PCR because there is a chance of inhibition or deficient amplification during PCR.
  If the heat‐shocked spores are not used immediately, we recommend keeping them in a –20°C freezer. Furthermore, after the PCR has been performed, we recommend putting the plate back at –20°C to preserve the genomic DNA for future use.
  We have used GoTaq (Promega) for PCR screening of up to 2 kb. When the downstream application, i.e., Sanger sequencing or Nanopore sequencing, requires a proofreading polymerase, we have had excellent results using VeriFi^™^ Polymerase (PCR Biosystems). With this enzyme and heat‐shock‐isolated DNA template, we have successfully amplified up to 5 kb.

## Basic Protocol 3 HIGH‐PURITY DNA ISOLATION AFTER MINI MYCELIAL MAT CULTURE

For whole genome sequencing (WGS), high‐quality genomic DNA is needed. Mycelial mats are a good source of fungal material for the isolation of genomic DNA. By inoculating the spore suspensions in liquid medium and using the 96‐well format to culture mycelial mats, enough fungal material can be obtained for the use of paramagnetic beads to isolate genomic DNA from *A. fumigatus*. This method produces high‐quality genomic DNA to prepare DNA libraries using the Nextera Flex kit (Illumina), creating libraries for paired‐end reads of 300 to 400 bp. The fungal material obtained after culturing can be immediately used or can be stored at –80°C for future DNA isolation.

### Materials

Liquid MEA medium (see recipe)
Spore suspension (in master plate; see Basic Protocol 1)
Liquid nitrogen
Extraction buffer (Tang et al., 1992; see recipe) + RNase A (at a final concentration of 150 µg/ml; Roche, cat. no. 10109142001)
5 M potassium acetate, 4°C
80% (v/v) ethanol (make fresh)
Clean MilliQ water

Multichannel reagent reservoirs, sterile
Multichannel pipet (100 to 1000 µl)
96‐deep‐well plate, 2 ml, pyramid bottom, 44‐mm height, sterile (Greiner, cat. no. 780271)
96‐deep‐well‐format lid, sterile
Micropore tape
Vortex
37°C incubator
2‐mm glass beads (Merck, cat. no. 1.04014.0500), autoclaved
Bead dispenser, compatible with 96‐well plates (Lab TIE, cat. no. BSP‐LT‐96‐2‐1‐40, or similar)
96‐well‐format cover seal (Greiner, cat. no. 381080) or adhesive PCR sealing foil sheets (Thermo Scientific, cat. no. AB‐0626)
Styrofoam container
Retsch Mixer Mill (MM 400, Retsch)
65°C water bath
Plate shaker (Heidolph, Titramax 1000) at 37°C
96‐well‐plate centrifuge, 4°C
Sera‐Mag SpeedBeads (carboxylate modified, hydrophilic; Cytiva, cat. no. 45152105050250; prepared according to Rohland & Reich, 2012)
Rubber hammer
Duct tape
Platform shaker
96‐well‐plate‐compatible magnet (Magnetic Stand‐96, Invitrogen, cat. no. 00833789)
96‐well PCR plate, sterile

**Figure 3:**
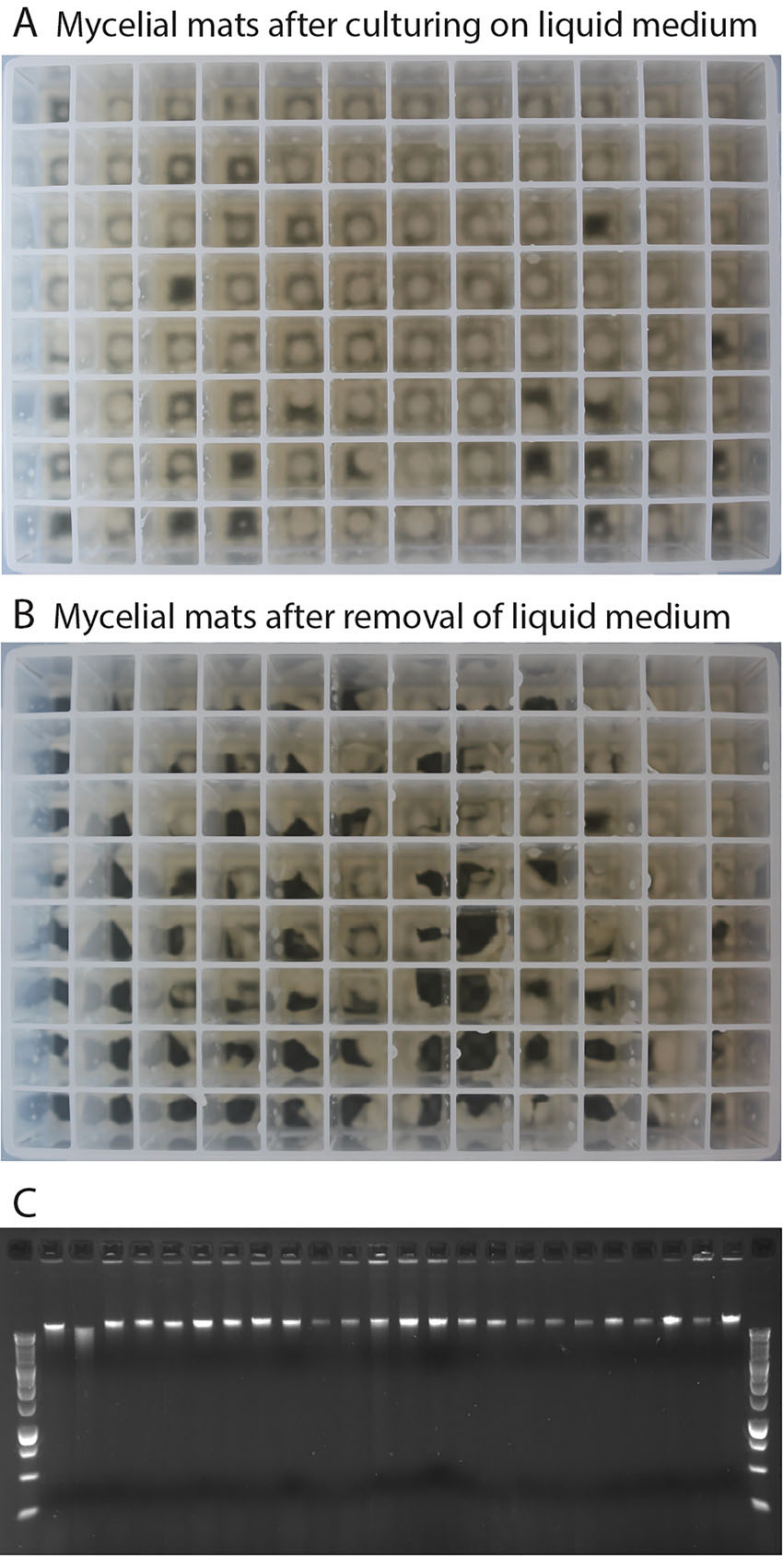
Mini mycelial mats and high‐molecular‐weight DNA isolation. To isolate higher‐quality genomic DNA for whole genome sequencing, we inoculate liquid medium to grow mycelial mats of *A. fumigatus*. (**A**) After <24 hr of incubation at 37°C, we observe that mycelial mats are formed at the top of the liquid medium. Mycelial mats at this stage display different shapes and can be seen in the center of the well; growing at the edges of the well, leaving a center with ungrown fungi; or growing toward one of the walls only. (**B**) Prior to disrupting the mycelia by grinding the mycelial mat, the liquid medium must be removed. After removing the liquid, the mycelial mats adopt different shapes at the bottom of the plate but are clearly visible. (**C**) Representative view of 24 isolated genomic DNA samples run on a 1% agarose–ethidium bromide gel. All samples show genomic DNA with a molecular weight >10 kb, compared with the higher band present in the 1‐kb ladder from Promega (leftmost and rightmost lanes).

1 Pour the liquid MEA medium (room temperature) into a sterile multichannel reagent reservoir.
2 Using a multichannel pipet, add 200 µl liquid MEA medium to each of the 96 wells of the sterile 96‐deep‐well plate.
3 Using the multichannel pipet, add 10 µl spore suspension (from master plate; see Basic Protocol 1).
4 Close the plate with a sterile 96‐deep‐well‐format lid, secure it with micropore tape, and vortex vigorously for 10 s.
5 Incubate in a 37°C incubator for a maximum of 24 hr or until mycelial mats are visible on the top of the medium.
  The time necessary for the mycelial mat to set might vary for different isolates, but make sure that green sporulation is not visible on top of the mat. The presence of spores will interfere with DNA extraction and affect the quality of the isolated DNA. If spores are visible, it is recommended to start the culture again.
6 Carefully remove the liquid medium with the multichannel pipet while avoiding touching the mycelial mat itself and discard the remaining medium according to biosafety regulations.
  The mycelial mat will remain at the bottom of the well but may also move to the walls; this is not a problem, but make sure that all liquid medium is removed.
7 Add 2‐mm glass beads (3 or 4) to each well using a bead dispenser compatible with 96‐well plates, close plate tightly using a 96‐well‐format cover seal or adhesive PCR sealing foil sheet.
  Once all the liquid medium is removed and the glass beads added, the plate can be stored at –80°C for future use. If this is done, close the plate tightly with either a silicone cover or a 96‐well aluminum sticky film.
8 To grind the mycelial mats to isolate DNA, freeze the plate containing the mycelial mats by placing it in a Styrofoam container filled with liquid nitrogen. Then, use a Retsch Mixer Mill to grind the mycelial mats to a powdery consistency for three rounds of 30 s at 30 Hz.
  Make sure the samples are frozen between the grinding rounds; for this, it might be necessary to remove and submerge the plate in liquid nitrogen. At the end of the grinding, it might be hard to see the sample powder, as it can be around or between the beads.
9 Add 300 µl extraction buffer + RNase A (at a final concentration of 150 µg/ml) and incubate for 15 min at 65°C in a water bath.
10 Remove the plate from the water bath and incubate for 1 to 1.5 hr at 37°C on a plate shaker at 500 rpm.
11 Put the plate on ice, add 75 µl ice‐cold 5 M potassium acetate per well, and incubate for 15 min on ice.
  If potassium acetate is not available, 300 µl ice‐cold 5M ammonium acetate can also be used.
  The plate can also be incubated overnight at 4°C to allow protein precipitation.
12 Centrifuge 5 min at 1000 × *g*, 4°C, in a 96‐well‐plate centrifuge.
13 Transfer 300 µl supernatant to a new 96‐deep‐well plate containing 300 µl Sera‐Mag SpeedBeads per well, close the plate tightly with a cover seal or foil sheet (use a rubber hammer if necessary), and seal it with duct tape.
  This prevents leaks in the following step.
14 Place the 96‐deep‐well plate on its side on a platform shaker at 50 rpm and incubate at room temperature for 1 hr.
  Efficient DNA binding to the paramagnetic beads requires sufficient time and agitation.
15 Centrifuge the plate briefly to make sure that all beads are in solution before the next step.
16 Put the plate on a 96‐well‐plate‐compatible magnet and allow the beads to settle for 5 min or until all the beads are down toward the magnet.
17 Remove the supernatant by inverting the plate, while still on the magnet, in one steady movement.
  This step can be done directly over the sink or over an intermediate container suitable for discarding the supernatant according to chemical regulations.
18 Using the multichannel pipet, add 500 µl freshly made 80% ethanol. Hammer the cover seal or foil sheet down to close the plate and mix carefully on the platform shaker, making sure that the beads come in solution. Vortex at mid‐speed if necessary.
19 Put the plate on a 96‐well‐plate‐compatible magnet and allow the beads to settle for 5 min or until all the beads are down toward the magnet.
20 Remove the ethanol by inverting the plate, while still on the magnet, in one steady movement.
  This step can be performed directly on the sink or in an intermediate container suitable for the disposal of the ethanol according to the chemical regulations.
21 Repeat steps 18 to 20 twice more.
22 After the last wash, remove as many ethanol traces as possible. Use the multichannel pipet if necessary and then let the beads carrying the DNA dry for 10 to 30 min at room temperature.
23 Once the bead pellet is dry, add 50 µl clean MilliQ water, shake horizontally on the platform shaker to resuspend the beads, and let the DNA dissolve in the water for 30 min at room temperature or overnight at 4°C.
24 Place the plate on the magnet until the beads settle and bind to the magnet.
  It is also possible to spin the beads down by centrifugation before placing them on the magnet to speed up this process.
25 Transfer ≤45 µl eluate containing genomic DNA to a new sterile 96‐well PCR plate. Make sure not to transfer the beads.
  To check the quality of the DNA, 2 µl is run on an agarose electrophoresis gel. If a 96‐well‐plate‐compatible system is used, the DNA can be loaded with a multichannel pipet, and the results can be visible after ∼1 hr, depending on the electrophoresis system.
26 Use 1 µl DNA for PCR quantification with a 96‐well‐plate‐compatible method.
  We use Quant‐iT PicoGreen dsDNA Reagent, from Thermo Fisher, or another method to quantify DNA in a 96‐well format.
  With this method, we obtain a range of concentrations from 2 to 10 ng/µl and observe genomic DNA fragments of 10 kb or higher (Fig. 3C). DNA can be immediately used to prepare DNA libraries. If the yield or quality of the DNA is nonexistent or extremely low, please refer to Table 1 for possible causes and solutions.

## REAGENTS AND SOLUTIONS

### Extraction buffer

2% (v/v) Triton X‐100
1% (w/v) SDS
100 mM NaCl
10 mM Tris·HCl, pH 8.0
1 mM EDTA, pH 8.0
Autoclave 15 min at 121°C
Store ≤1 month at room temperature (short‐term storage) or ≤6 months (longer‐term storage) at 4°C
  This recipe is from Tang et al. (1992).

### Malt extract agar (MEA) medium

3% (w/v) MEA (Merck, cat. no. 1053910500)
1.5% (w/v) microbiological agar
1 mg/L CuSO_4_
Autoclave 15 min at 121°C
Store ≤1 week at 55‐60°C in an incubator
  If the medium is not going to be used immediately, let it solidify at room temperature and melt it in a microwave prior to use.

### PBS‐0.05% Tween‐20 buffer

10 mM Na_2_HPO_4_
137 mM NaCl
1.8 mM KH_2_PO_4_
2.7 mM KCl
0.05% (v/v) Tween‐20
Adjust to pH 7.3‐7.4 with 1 M HCl or 1 M NaOH if needed
Autoclave 15 min at 121°C
Store ≤6 months at room temperature
  This recipe is from a prior Current Protocols article (Fraczek et al., 2019).

## COMMENTARY

### Critical Parameters

When working with sporulating fungi, particularly *A. fumigatus*, it is essential to reduce the chances of cross‐contamination. When inoculating colonies from a cultured plate into a 96‐well mini slant format (Basic Protocol 1), it is vital to use a tungsten needle rather than inoculation loops. We have also used fine (2‐mm) cotton swabs, and they can work well if not overloaded with spores. If using swabs, gently touch the colony on the starting plate with one side of the swab and inoculate the mini slant. If spore suspensions are used and they are already arranged in a 96‐well format, use 1 to 2 µl to inoculate the mini slants using a multichannel pipet for small volumes (0.5 to 10 µl).

It is essential to include one or more negative growth controls in your 96‐well mini slant plate arrangement (Basic Protocol 1). These negative controls are mini slants that contain medium but are not inoculated, and they should look clear after incubation at 37°C. Treat them as any other sample throughout the procedure; they should not produce any PCR product or yield genomic DNA. If they do show unexpected growth in the mini slants, this is an indication of cross‐contamination, and the procedure should be stopped and started over.

To obtain mycelial mats (Basic Protocol 3), it is also critical that after incubation at 37°C, no spores are visible on the mats. If this happens, which is easy to spot because the mat becomes grayish or shows dark green areas within, it is better not to use it or to be aware that the genomic DNA yield and purity may be reduced. To optimize this step, it is important not to exceed 24 hr of incubation at 37°C. It is recommended to test the optimal incubation time for your isolates and the inoculation density; it is possible that after 16 to 20 hr, the mats are ready to be used. In this sense, a smaller mat does not necessarily produce a reduced amount of DNA because the amount of DNA depends greatly on how well the mats are ground and how many cells are exposed to the lysis/breaking buffer. Be aware that more is not always better.

This set of protocols is likely applicable to other sporulating fungi, with adjustments to incubation amounts/times depending on the biology of each species. We have not tested these protocols in nonsporulating fungi, but in principle, if a mycelial mat can be obtained by inoculating the liquid medium with a different starting material, then Basic Protocol 3 can also be used to generate the mycelial mats.

The efficiency of the protocols and procedures described here depends greatly on the use of 96‐well plates and multichannel pipets. Be systematic in how you arrange your samples, make sure the pipet tips are changed when needed, and work neatly to reduce cross‐contamination.

### Troubleshooting

Please see Table 1 for potential problems and their solutions.

### Understanding Results

Basic Protocol 1 allows us to scale up the culture of *A. fumigatus* into a 96‐well format. The downstream procedures (Basic Protocols 2 and 3), also described here, can be selected depending on the desired screening method and/or the research question (Fig. 1). This culture method can also be used for other applications, for example, to perform phenotypic analysis on a solid substrate in a 96‐well format (Cánovas et al., 2017).

The mini slant culture protocol (Basic Protocol 1) describes the preparation and culture of 96 mini slants, each of them containing a single inoculum or isolate of *A. fumigatus*. The mini slants are prepared using any fungal medium in a deep (2‐ml) 96‐well plate. The area of all of the mini slants is enough to allow the growth of the fungus and the production of a good number of spores after 48 hr of incubation at 37°C (Fig. 2A). The spore suspensions obtained after harvesting can be used for different purposes, including (1) the preparation of PCR‐quality DNA (Basic Protocol 2) and (2) the preparation of mini mycelial mats, from which high‐purity genomic DNA can be obtained (Basic Protocol 3).

To prepare PCR‐quality DNA, Basic Protocol 2 uses a fast heat‐shock method to obtain DNA from *A. fumigatus* (see Current Protocols article: Fraczek et al., 2019). Using this heat‐shock procedure, we have been able to obtain clear evidence that our 96‐well‐format culture method is clean and reliable and that, when performed correctly, there is no contamination between adjacent samples. To confirm the above, we took a 96‐well plate and, using a tungsten needle, inoculated 90 wells with a wild‐type isolate of *A. fumigatus* (AfIR974) (Sugui et al., 2011). In four remaining wells, a stable transformant isolate containing the hygromycin phosphotransferase (*hph*) gene, conferring hygromycin resistance, was inoculated. The two remaining wells were not inoculated (Fig. 2B and 2C). When PCR amplifying the coding region of *hph* was performed, we observed amplification of this gene only in samples from the four wells with the stable transformant isolate containing *hph*, and not in samples in any other wells (Fig. 2D). To ensure genomic DNA was successfully isolated from all wells and that the absence of bands was not due to the lack of template, we amplified calmodulin (*cmd*), a housekeeping gene, using the primers cmd5: 5’‐CCGAGTACAAGGARGCCTTC‐3’ and cmd6: 5’‐CCGATRGAGGTCATRACGTGG‐3’ (Hong et al., 2005). The *cmd* PCR showed a band of the expected size in all the wells, except for the two negative‐control wells, indicating that genomic DNA was present in all 94 inoculated wells (Fig. 2E). PCR products obtained with this method can be used for agarose gel electrophoresis to screen genotypes based on amplicon size, Sanger sequencing to determine the allelic variation of single isolates, or nanopore sequencing to identify genomic variation within a given population of spores.

**Figure 4:**
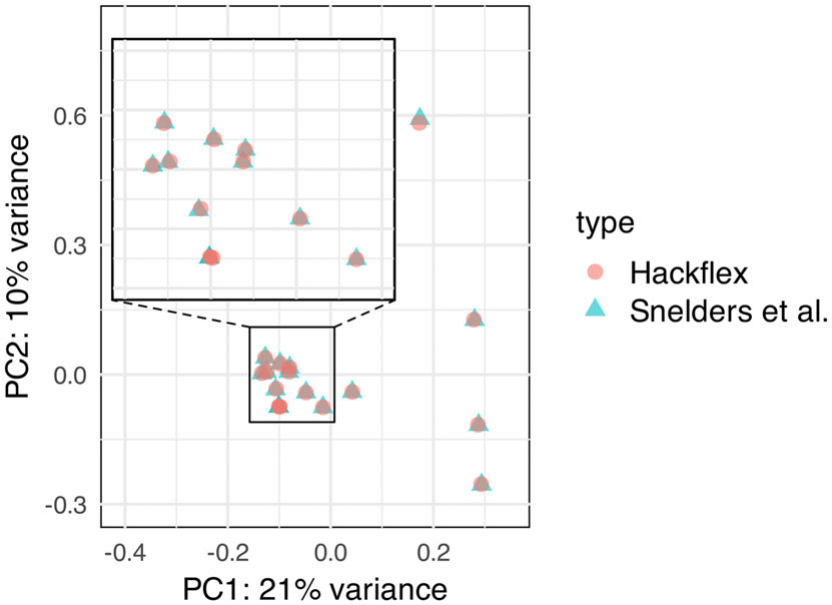
Comparison of genome‐wide variants. Principal component analysis (PCA) of genome‐wide variants across 15 *A. fumigatus* samples. Salmon‐colored circles represent samples prepared according to the approach described here. Teal triangles represent samples prepared with traditional genomic DNA library preparation. Overlap between circles and triangles represents genomic similarity between analysis methods. The inset shows a close‐up of a subset of samples.

Finally, our mini mycelial mat culture protocol (Basic Protocol 3 and Fig. 3) describes the production of enough non‐sporulating fungal tissue in a 96‐well format to isolate high‐molecular‐weight genomic DNA (Fig. 3A). After removing the liquid medium, the mycelial mats adopt different shapes at the bottom of the tubes (Fig. 3B). Once the mats are ground and the cells disrupted, we use paramagnetic beads to isolate genomic DNA. This method allows us to obtain highly pure genomic DNA of high molecular weight (>10 kb; Fig. 3C). The DNA obtained here can also be used as a template for PCR; however, due to the high concentration, dilution is necessary before its use as template DNA. The most significant aim of this protocol is to obtain high‐quality genomic DNA that can be used to prepare DNA libraries for paired‐end reads for use in short‐read WGS. To verify that this protocol would allow WGS of individual *A. fumigatus* isolates rapidly and cost effectively, we processed our DNA extracts with the Hackflex protocol (Gaio et al., 2022). Comparison of the variants identified with these samples against gold‐standard DNA sequencing (Snelders et al., 2024) showed high congruency of results (Fig. 4 and Supporting Information, File 1). So long as sequencing depth over 15× is achieved, the variants called between this method and the gold standard are >99% similar (average of 226 discrepancies out of 32,655 variants). It should be noted that when coverage is lower than 15×, there can be significant discrepancies between the results.

### Time Considerations

The value of this set of protocols is in the time and spaced saved, allowing a high‐throughput setup based on the use of a multichannel pipet and 96‐well plates. These protocols enable processing and analysis of a high number of samples that were previously simply not possible with the currently available protocols.

To make a 96‐well mini slant deep‐well plate (Basic Protocol 1), ∼30 min is needed, which includes the time necessary to dry the slants. The time required for inoculation will depend on the starting material: for spore suspensions or single colonies grown on plates, roughly 30 to 45 min is needed. For *A. fumigatus*, we use a standard 48‐hr incubation to obtain sufficient spores. To collect spores, 30 min is needed, which includes a 15‐min incubation step and the transfer to a new plate. The quick DNA isolation protocol (Basic Protocol 2) takes 1 hr maximum, depending on the final temperature used to cool down the spore suspension. Setting up a 96‐well‐plate PCR reaction can take up to 30 min, and a standard PCR runs for ∼2 hr depending on the polymerase used and the length of the PCR product.

To set up the liquid cultures for the growth of the mycelial mats (Basic Protocol 1), 10 min maximum is required. The inoculation can take as little as 5 min if the spore suspensions are already in a 96‐well format. The plate containing the mycelial mats is grown for a maximum of 24 hr. The isolation of high‐purity DNA can take up to 5 hr, which includes two incubations of 1 hr each. It takes ∼1 hr to quantify the DNA if a 96‐well‐format‐compatible method is used. To check the quality of the isolated DNA, an electrophoresis in agarose gel is advised; this step can take up to 1.5 hr depending on the electrophoresis system and the running conditions.

### Author Contributions

**Francisca C. Reyes Márquez**: Conceptualization; data curation; formal analysis; investigation; methodology; project administration; validation; writing—original draft. **Ben Auxier**: Data curation; investigation; methodology; software; validation; visualization; writing—review and editing. **Bo Briggeman**: Formal analysis; investigation; methodology; writing—review and editing. **Eveline Snelders**: Funding acquisition; project administration; resources; supervision; writing—review and editing.

### Conflict of Interest

The authors declare no conflict of interest.

## Supporting information


**File 1** Hackflex comparison data.

## Data Availability

The data, tools, and material (or their source) that support the protocols are available from the corresponding author upon reasonable request. Analysis code for genomic comparisons can be found at https://github.com/fungalsnelderslab/hackflex_96wellsformat. Raw short‐read DNA sequencing data can be found under project ERP166589.
